# Respiratory Health of Pacific Youth: An Observational Study of Associated Risk and Protective Factors Throughout Childhood

**DOI:** 10.2196/18916

**Published:** 2020-10-21

**Authors:** El-Shadan Tautolo, Conroy Wong, Alain Vandal, Shabnam Jalili-Moghaddam, Emily Griffiths, Leon Iusitini, Adrian Trenholme, Catherine Byrnes

**Affiliations:** 1 AUT Pacific Health Research Centre Faculty of Health & Environmental Sciences Auckland University of Technology Auckland New Zealand; 2 Department of Respiratory Medicine Middlemore Hospital Counties Manukau District Health Board Auckland New Zealand; 3 Department of Biostatistics University of Auckland Auckland New Zealand; 4 Paediatric Department Faculty of Medical & Health Sciences University of Auckland Auckland New Zealand

**Keywords:** respiratory, Pacific Islands, public health, risk, growth and development, youth

## Abstract

**Background:**

Respiratory disease is the third most common cause of death in New Zealand, with Pacific people living in New Zealand bearing the greatest burden of this type of disease. Although some epidemiological outcomes are known, we lack the specifics required to formulate targeted and effective public health interventions. The Pacific Islands Families (PIF) birth cohort study is a study that provides a unique source of data to assess lung function and current respiratory health among participants entering early adulthood and to examine associations with early life events during critical periods of growth.

**Objective:**

This paper aims to provide an overview of the design, methods, and scope of the *Respiratory Health of Pacific Youth Study*, which uses the overall PIF study cohort aged 18-19 years.

**Methods:**

From 2000-2019, the PIF study has followed, from birth, the growth, and the development of 1398 Pacific children born in Auckland, New Zealand. Participants were nested within the overall PIF study (at ages 18-19 years) from June 2018, and assessments were undertaken until mid-November 2019. The assessments included respiratory and general medical histories, a general physical examination, assessment of lung function (forced expiratory volume and forced vital capacity), self-completed questionnaires (St George’s Respiratory Questionnaire, European Quality of Life 5 Dimensions-3 Level, Epworth Sleepiness Scale for Children and Adolescents, and Leicester Cough Questionnaire), blood tests (eosinophils, Immunoglobulin E, Immunoglobulin G, Immunoglobulin A, Immunoglobulin M, and C-reactive protein), and chest x-rays. Noninferential analyses will be carried out on dimensionally reduced risk and protective factors and confounders.

**Results:**

Data collection began in June 2018 and ended in November 2019, with a total of 466 participants recruited for submission of the paper. Collection and collation of chest x-ray data is still underway, and data analysis and expected results will be published by November 2020.

**Conclusions:**

This is the first longitudinal observational study to address the burden of respiratory disease among Pacific youth by determining factors in early life that impose long-term detriments in lung function and are associated with the presence of respiratory illness. Identifying risk factors and the magnitude of their effects will help in adopting preventative measures, establishing whether any avoidable risks can be modified by later resilient behaviors, and provide baseline measurements for the development of respiratory disease in later adult life. The study results can be translated into practice guidelines and inform health strategies with immediate national and international impact.

**International Registered Report Identifier (IRRID):**

DERR1-10.2196/18916

## Introduction

### Background

Respiratory disease is the third most common cause of death in New Zealand [1,2], with 69,000 hospitalizations per year. Hospitalizations are 5.1 times higher and mortality is 2.7 times greater in the most deprived geographical areas compared with the least deprived areas [3]. Pediatric hospital admissions for bronchiolitis, asthma, wheezing, and viral pneumonia have increased since 2000 to over 21,000 per year [4]. In the same period since 2000, hospitalizations in all ages for bronchiectasis increased by 30%, with a doubling of deaths [3]. Over 28,000 people in New Zealand are estimated to have severe chronic obstructive pulmonary disorder (COPD), with up to 15% of the total population suspected to have the disease [3,5]. This may partly explain why this rise in respiratory disease is strongly demonstrated in South Auckland [4], a geographical area with very high levels of deprivation, particularly among its large Pacific and Māori populations.

Pacific people (Samoan, Tongan, Cook Islands Māori, Niuean, and Tokelauan) living in New Zealand are the fourth largest population group, and the third largest living in Auckland, New Zealand’s biggest city [6]. Of the Pacific population resident here, 21% live in South Auckland (Manukau District) [7], and 76% of these people live in the most deprived areas [8]. Of all ethnic groups, Pacific people bear the greatest burden of respiratory diseases [2], and across all age groups, their hospitalization rates for these illnesses are 2.6 times higher than those for other ethnic groups [2,3,9]. Relative risks for the Pacific population range between 1.7 and 18.2 for asthma [10], bronchiectasis [10,11], bronchiolitis [12], pneumonia [13], COPD [14,15], and obstructive sleep apnea [16], compared with non-Pacific people. This represents a significant health disparity for Pacific people, who have the highest proportion of individuals in the 15- to 24-year age bracket [17], and is expected to constitute 10% of the population and 12% of the working-age population by 2026 [18].

Lung development starts in utero with substantial structural development and continues through early childhood, with the alveoli likely increasing in number, size, and complexity through adolescence [19,20]. Maximum lung volumes are reached around 20 to 22 years of age for males and slightly earlier (around 18-20 years) for females [21]. This represents a 30-fold increase in lung volume and a 20-fold increase in gas-exchanging surface area with at least a doubling of airway length and diameter. Nevertheless, after peaking in early adulthood, lung function gradually declines with age in healthy individuals because of factors including loss of lung elasticity, the decline in respiratory muscle strength, and reduced alveolar surface area [22].

Current scientific theory indicates that many adult respiratory diseases arise from early events during the period of rapid growth from infancy through childhood [19,23]. The impact of respiratory events early in life appears to be two-fold: (1) early insults may prevent attainment of peak lung function with a subsequent decline from a lower peak level and/or increase the rate of rapid decline and (2) early disease increases susceptibility to developing a later disease. These factors may predispose to the development of chronic lung diseases such as asthma, COPD, and bronchiectasis in adulthood [24].

### Reduced Lung Function

Intrauterine growth restriction is a risk factor for reduced lung function during infancy [25], childhood [26], and adulthood (using self-reported birth weight) [27]. In-utero smoke exposure is associated with reduced lung function at birth [28], early childhood [29], and early adulthood [30,31], and is similarly associated with accelerated lung function decline [32]. Living with a smoker up to the age of 18 years increases the risk of cough and sputum production as adults [33]. In addition, asthma in childhood is associated with lower lung function in adulthood [34-36] and a more rapid decline in lung function [37].

Once airway restriction has occurred, the composite picture from other overlapping studies in healthy and asthmatic populations suggests that lung function centiles tend to track with time [38,39]. First, cohort studies in Tucson (n=826) [40], Perth (n=243) [41], Sydney (n=10,898) [42], Manchester (n=690) [43], and the Netherlands (n=838) [44] have shown that the majority of early infant wheezers have reduced lung function at school age. Second, these studies have also shown that from infancy to childhood (n=95) [41], infancy to early adulthood (n=169) [39], across childhood [41-43], and into early adulthood from 9 years (n=646) [45] and 11 years (n=600) [38], those with low lung function on the first assessment remained in the lower centile. A large community study on atherosclerosis risk (n=15,536) described an increased risk of COPD in those with accelerated lung function decline (excluding individuals who smoke) [46].

### Early Disease Predisposing to Later Disease

In seminal research, Barker et al [47,48] reviewed death certificate data across several communities in England, showing that an area with a high infant pneumonia mortality rate had a high COPD mortality rate 15 years later, suggesting an individual-level association. Further studies indicate that the number of lower respiratory tract infections in childhood seemed to predict the presence of obstructive airway disease and ventilatory impairment in adults [49,50]. Childhood pneumonia is described as a sentinel event in 28% to 42% of adult populations with bronchiectasis [51-53], with 60% to 80% reporting wet cough since childhood [52,54].

In the Dunedin Study birth cohort, 26.9% of the participants had continuing symptoms of asthma, with half persistent and half recurring in early adulthood [38]. Admission for bronchiolitis or pneumonia when <5 years of age was associated with an increased risk of doctor-diagnosed asthma and increased medication use [36,55], which in the European Community Respiratory Health Survey was shown to be further exacerbated by smoke exposure [36]. A history of asthma in childhood was also associated with a 5.2 to 12.5 increased risk of COPD [5,56]. However, most of these studies have sampled adult populations and relied on individual recall of childhood events. Asking a participant with a current respiratory diagnosis whether they had an infection in childhood may well introduce bias and, given the prevalence of respiratory infections in childhood, may not be discerning.

Fewer studies have examined the impact of risk or protective factors such as vitamin deficiencies, physical activity levels, and breastfeeding on respiratory function and later respiratory disease. Despite early pathological studies that indicated that the alveolar structure was complete by 2 years of age, recent magnetic resonance imaging suggests that the alveoli continue to develop into young adulthood [57-59]. This may widen the possibility of ongoing damage with new insults but theoretically also implies that resilient behaviors may improve later lung growth. In one study, breastfeeding was found to aid lung growth and was associated with improved forced vital capacity (FVC) at the age of 10 years but not 18 years [60,61].

Vitamin deficiencies (vitamins A, D, and E) seem to have a greater effect on alveolar development than on airway development [62,63]. Maternal vitamin A supplementation was shown to increase FVC and forced expiratory volume in 1 second (FEV1) among 1894 children aged 9 to 13 years living in Nepal (a population with high vitamin A deficiency) compared with children whose mothers had received a placebo [62]. A study examining dietary antioxidants among 243 healthy, nonsmoking students in the United States (mean age of 18.3 years, SD 1.95) showed that vitamin C and magnesium intake was associated with higher lung function in these college students [64]. Breastfeeding [65-68], immunizations [69,70], and adequate vitamin D levels [71-74] are all associated with fewer early childhood infections, which may protect lung development.

There are some known gender differences in respiratory disease patterns. Hospitalization rates for total respiratory admissions are higher in boys than girls when less than 15 years of age but become more common in women than in men as adults [75]. Hospitalization rates for asthma have a rate ratio of 2.75 for children (<15 years of age) when compared with adults (30-64 years of age), but there are clear gender differences. Girls with a rate ratio of 0.78 were compared with boys, but women with a rate ratio of 1.86 were compared with men. Similarly, medicated asthma in 2016 to 2017 was seen in 11.3% of girls and 17.2% of boys, whereas it was present in 14% of women but only 9.9% of men. Hospitalization for bronchiolitis, bronchiectasis, and pneumonia occurs more frequently in boys (rate ratios for girls between 0.64 and 0.98) [75]. Although hospitalization rates for COPD were significantly higher in women than men by 45 to 64 years of age, mortality was similar between genders, but mortality for women with bronchiectasis was higher than men [76]. Smoking rates also differed, with 28% of Pacific men and 22% of Pacific women smoking. In addition, Pacific people have the most rapid transition from experimentation to regular smoking of 2.7 years when compared with other community groups [77].

The Pacific Islands Families (PIF) study birth cohort is an ongoing observational study of the health and development of a birth cohort of children of Pacific ethnicity and their parents. The selected findings from the study included a high immunization uptake (89%) among the cohort over the first 2 years of life [78]. At 4 years of age, many children in the cohort and their mothers had poor basic oral hygiene (34% of mothers were brushing ≤1/day and 50% had either never seen a dentist or had not visited one in the last 5 years, 47% of the children brushed ≤1 time per day, and 47% had no adult assistance with brushing) [79]. Moreover, 57% of these children were routinely snacking or drinking immediately before bed, which substantially increased their health risk [79]. Parental smoking prevalence and secondhand smoke exposure among the cohort aged 11 years indicated very high prevalence rates (33% of mothers and 40% of fathers) [80], resulting in about 50% of families with at least one parent who smoked, and 25% with both parents who smoked [81]. This suggests that environmental smoke exposure is a significant health risk for children from the cohort living in these households. To date, there has been no formal respiratory assessment undertaken among the cohort. This paper provides an overview of the design, methods, and scope of *Respiratory Health of Pacific Youth*, a retrospective study of early childhood events and their impact on current respiratory status nested within the overall PIF study (at ages 18-19 years).

### Study Objectives

This study will address 3 objectives: (1) estimate the effect of early life (eg, birthweight, antenatal smoke exposure, postnatal smoke exposure) and childhood risk factors (eg, allergies, dwelling conditions from the first 2 years of life, child smoking at 14 years) on peak lung function attainment and respiratory outcomes in Pacific youth aged 18 to 19 years; (2) determine modifiable childhood risk and protective factors; including breastfeeding, immunization, and nutrition during the first 2 years of life; exercise at ages 4, 11, and 14 years; peak flow at ages 6 and 9 years; respiratory infections, respiratory condition–related hospital admissions, and reported breathing problems in the first 2 years of life; and asthma in childhood) on lung function attainment and respiratory outcomes in Pacific youth aged 18 to 19 years; and (3) estimate the population attributable fraction and population avoidable fraction of modifiable early life risk factors and childhood resilience factors on these outcomes. We hypothesize that at age 18 to 19 years, (1) early life risks result in poorer lung function and respiratory outcomes in early adulthood in Pacific youth and (2) protective or resilience factors throughout childhood moderate the impact of these early life risks on these poorer lung function and respiratory outcomes in early adulthood.

## Methods

### Study Design

The PIF birth cohort study is a multidisciplinary study [82] tracking the health and development of 1398 Pacific children born at Middlemore Hospital, South Auckland, New Zealand, in 2000. A child was defined to be of Pacific Islands ethnicity if at least one parent self-identified as being of that ethnicity and only eligible if at least one parent was a permanent resident of New Zealand [82]. The PIF study provides a unique source of data for research on growth, development, and psychosocial functioning at critical developmental stages within the family environment. The size of the cohort was chosen to enable the generation of findings that were specific to the predominant Pacific groups residing in New Zealand (Samoan, Tongan, and Cook Islands Māori) [83]. Assessments and interviews were conducted at 6 weeks of age, then at 1, 2, 4, 6, 9, 11, 14, and 17 years of age. This study collected cross-sectional data on respiratory outcomes from the cohort over 2018 to 2019, when they were aged 18 to 19 years. All antecedent data collected at previous measurement waves will be available for inclusion in the analysis where appropriate. Biological sex and gender stratification will also be considered in the analysis of respiratory data.

### Study Population

In recognition of attrition and residential mobility of the initial PIF cohort (n=1398, 681 females and 717 males, to n=954, 463 females and 468 males) by age 14 years, we anticipated achieving a sample size of 750 youths from the original cohort. Since June 2018, an assessment of Pacific youth aged 18 to 19 years was initiated. The only exclusion criteria were (1) exclusion of cohort members whose current sickness would prohibit them from producing maximal effort during lung function testing and (2) exclusion of cohort members resident outside of Auckland, as assessments could only be undertaken in Auckland.

### Study Procedures

Ethical approval for this study was obtained from the Central Health and Disability Ethics Committee on May 24, 2018 (reference 18/CEN/24). Written informed consent was obtained from the youth to participate in an assessment involving a series of physical and clinical assessments and self-administered web-based questionnaires. Arrangements were made for 2 research assistants of Pacific ethnicity to transport participants to and from a clinic set up for the respiratory assessments at the University of Auckland (Tāmaki campus) and Ascot hospital where chest x-rays and blood tests were performed. The youth were thanked with a gift voucher for their participation. The data sets used during this study are available from the corresponding author upon reasonable request.

### Outcomes: Clinical Assessments

The primary outcome of this study was the FEV1 Z-score standardized for height, gender, and age (American Thoracic Society/European Respiratory Society criteria using Global Lung Initiative reference values) as a continuous variable. The FEV1 Z-score will be dichotomized to an indicator of a Z-score <−1.64 for estimating the population attributable risk.

Other clinical assessments involve the documentation of participants’ respiratory and general medical histories, medications, a clinical examination (respiratory rate, cardiovascular, and ear, nose, and throat examinations), and a Bacillus Calmette–Guérin vaccine scar presentation, clubbing severity (categorized into mild, moderate, or severe), tonsil score (according to the Brodsky and Friedman Scales [84]), and Mallampati score (a visual assessment of the space between the base of the tongue and the roof of the mouth that is an independent predictor of obstructive sleep apnea [85,86]). In addition, social behavior questions on work and sleep schedules, housing conditions, and exercise routines were asked. Participants were also asked about their caffeine, alcohol, tobacco, and drug intake.

The number of pulmonary exacerbations in the previous 12 months was documented. Symptom severity (cough, sputum color, and dyspnea) was recorded on validated 5-point scales [87,88], including (1) cough severity rated on a Likert-like symptom scale, (2) dyspnea severity rated according to the Modified Medical Research Council Dyspnea Scale [89], and (3) sputum color rated according to a Bronkotest color chart [90].

In addition, oxygen saturation (SPO_2_; Medtronic, Nellcor PM10N) and spirometry pre- and postsalbutamol (EasyOne Air Spirometer, NDD Medical Technologies) were assessed. Spirometry was performed according to American Thoracic Society standards [91] with predicted values from Global Lung Initiative reference values [92]. Baseline forced expiratory measurements were performed until 3 good quality, repeatable measures were obtained (FVC and FEV1 both within 0.15 l). This was followed immediately by the administration of 400 µg of salbutamol using a meter dose inhaler through a volumetric spacer device, and reversibility was tested by using spirometry after 15 min.

Nonfasting blood tests were completed at Ascot Hospital by Labtests, a pathology laboratory service accredited by the International Accreditation New Zealand. Trained phlebotomists drew 10 mL of blood for testing the levels of Immunoglobulin E, Immunoglobulin G, Immunoglobulin A, and Immunoglobulin M, eosinophils, and C-reactive protein. A further 6 mL of serum was stored at Middlemore Hospital tissue bank for future analysis of biomarkers; a separate consent for this analysis was obtained.

Chest x-rays (posteroanterior and lateral) were performed by Ascot Radiology. The radiation dose is 0.02 millisieverts with background radiation in comparison being 3 to 4 millisieverts, which is equivalent to 3 days of usual background radiation exposure. No chest x-rays were taken if a participant was pregnant. Chest x-rays were scored by 2 scorers using the Brasfield scoring system. The Brasfield system [93] consists of scoring chest x-rays using graded responses for 5 specific aspects: air trapping (scored 0-4), linear markings (bronchial wall thickening; 0-4), nodular cystic lesions (bronchiectasis; 0-4), large lesions (atelectasis and pneumonia; 0-5), and general severity (0-5). A score of 25/25 represents normal lungs, with numbers detracted for changes seen with lower scores representing more severe disease.

### Outcomes: Physical Measurements

Body size and composition measurements included height (Seca 213), weight (Tanita BC545), waist circumference with a nonstretchable tape, standing hand-to-foot bioimpedance analysis (ImpediMed Single Frequency 50 kHz Bioimpedance Analyzer, Tanita BC545), and blood pressure using an automated sphygmomanometer (Omron Auto Blood Pressure monitor IA2, Omron Healthcare) with appropriate cuff sizes.

Anthropometric and blood pressure measurements were repeated until 2 measurements were recorded within a predetermined tolerance (weight ±0.5 kg, height and waist ±0.5 cm, and systolic and diastolic blood pressure ±10 mm Hg). BMI was calculated as weight in kg/height in meters squared, and prevalence of obesity, overweight, and thinness were derived, standardized for age and gender using the Cole cutoffs [94].

### Outcomes: Questionnaires

Participants self-administered 4 questionnaires on a tablet computer with the research nurse present to assist as necessary: St. George’s Respiratory Questionnaire (SGRQ), European Quality of Life 5 Dimensions-3 Level, Epworth Sleepiness Scale for Children and Adolescents (ESS-CHAD), and the Leicester Cough Questionnaire (LCQ).

The SGRQ [95,96] includes 56 items across 3 domains: symptoms, activity, and impact. Component scores from each domain and a total score between 1 and 100 will be examined. Higher scores indicate poorer health.

The European Quality of Life-5 Dimensions is a generic measure of self-reported health status [97]. Health status was measured in terms of 5 dimensions: mobility, self-care, usual activities, pain/discomfort, and anxiety/depression, as well as a Visual Analogue Scale.

ESS-CHAD [98], a validated measure of daytime sleepiness for use with children and adolescents [99], was used to indicate the possibility of obstructive sleep apnea. A score of 13 to 15 represents a moderate risk and a score of >15 represents a severe risk for obstructive sleep apnea. The LCQ evaluates the impact of cough on the quality of life [100].

Any abnormal results were discussed on a case-by-case basis between the research nurses conducting the assessments and the coinvestigators of the study with backgrounds in respiratory medicine. In cases of incidental findings or results of concern, a referral letter was addressed to their general practitioner, along with notifying general practitioners of their patients’ involvement in the study and that the chest x-ray and blood tests of their patients will be available from the standard clinical information portal (Concerto).

### Data Analysis: Statistical Considerations

A research electronic data capture (REDCap) database (hosted on the Auckland University of Technology server and fully compliant with International Organization for Standardization standards and international data management) was set up to capture all data. REDCap is a web-based system that can be used for direct data input or secondary input from paper-based clinical record forms and questionnaires.

The key objective of the analysis was to obtain causal effect estimates of risk factors and modifiable protective factors on respiratory outcomes, conditional on preexisting risk factors and confounders. The analyses will be carried out in 3 stages: (1) dimensional reduction of the covariates, (2) causal modeling of the risk and protective factors, and (3) estimation of the population attributable and avoidable risks associated with the risk and protective factors.

### Dimensional Reduction

A technical challenge to overcome in this study is the large dimensionality of the covariates involved, potentially leading to overfitting. To alleviate this problem, we will apply dimensional reduction techniques to the covariates, taking care to maintain interpretability where necessary, especially with regard to population attributable and avoidable risks. The dimensional reduction will proceed using variations in sliced inverse regression (SIR) [101] from the primary outcome. The variations considered are specifically adapted to longitudinal covariate data [102,103] and categorical covariate data [104]. This approach to dimensional reduction will determine, in practice, a set of linear combinations of the covariates corresponding to confounders or to a specific risk/protective factor or specific respiratory conditions that best explain the primary outcome (such linear combinations can be interpreted in a manner similar to factors in a factor analysis). In this fashion, we will reduce the dimensionality of the set of all confounders, the main purpose of this step, and may be able to reduce the dimensionality of a specific risk or protective factors for which we have longitudinal data (eg, exercise) or several simultaneous measures (eg, nutrition), preventing overfitting. Secondary outcomes will be analyzed using the dimensionally reduced covariates obtained from the SIR on FEV1 and will not themselves be the object of an SIR to promote interpretability.

### Causal Inference

With *Y_i_* representing the outcome of interest (primary outcome FEV1 Z-score or any of the secondary outcomes) in participant *i*, the causal model we will consider is a simple extension from Robins et al [105].

*E*[*Y_i_*|*X_i_*, *R_i_*, *Z_i_*, *C_i_*] = α'*R_i_* + β'*X_i_* + *γ*'(*X_i_*:*R_i_*) + *g*(*C_i_*) + *h*(*Z_i_*) **(1)**

where α, β, and γ are the causal parameter vectors of interest; *X_i_* and *R_i_* denote the modifiable protective and risk factors, respectively, and *X_i_*:*R_i_,* their interaction; *C_i_* denotes the confounders and *Z_i_* the early life respiratory conditions; and *g* and *h* are semiparametric functions (typically smoothes or simply affine functions). Parameters, α, β, and γ are estimated as fully adjusted causal relationships using a two-stage estimation technique from Robins et al [105], which relies on the first-stage estimated conditional expectations *Ê* [*R_i_*|*C_i_*, *Z_i_*], *Ê* [*X_i_*|*C_i_*, *Z_i_*], and *Ê* [X_i_:*R_i_*|*C_i_*, *Z_i_*]. These latter quantities are estimated using appropriate linear, logistic, and multinomial models and consist of the multivariate equivalent (from a data analytical point of view) of propensity scores (the distinction is that propensity scores apply when a single risk factor or treatment is involved). Causal inference is thus based on a semiparametric regression model that adjusts for multivariate propensity scores; the preferred method of propensity score inverse weighting as used in a study by Austin [106] is not applicable in this case, precisely, due to the multiplicity of risk and protective factors.

For categorical outcomes, a logit link function is applied to the right-hand side of the above equation (see also the Inferential Setting section). The rest of the data analytical approach follows without modification.

We note that the estimation of α addresses hypothesis 1, whereas the estimation of β and the interaction term parameter γ addresses hypothesis 2. The causal model considered above treats early life respiratory conditions on an equal footing with confounders. We will also attempt the analysis by simultaneously fitting the following components, creating a mediation analysis where early life risk factors may affect later respiratory outcomes:

Direct path: *E*[*Y*_*i*|*X_i_*, *R_i_*, *C*_*i*] = *α*_0_'*R_i_* + *β*_0_'*X_i_* + *γ*_0_' (*X_i_*:*R_i_*) + *g*_0_(*C_i_*) **(2)**

Mediated path: *E*[*Y*_*i*|*X_i_*, *Z_i_*, *C*_*i*] = *ξ*'*Z_i_* + *β*_1_'*X_i_* + *g*_1_(*C_i_*)

*E*[*Z*_*i*|*X_i_*, *R_i_*, *C*_*i*] = *α*_2_'*R_i_* + *β*_2_'*X_i_* + *γ*_2_' (*X_i_*:*R_i_*) + *g*_2_(*C_i_*)

In our experience, mediated analyses such as equation 2 can easily become intractable numerically. We are likely to resort to the mediation model when considering specific early childhood conditions with reasonably high prevalence, such as asthma, as opposed to a full set of early childhood conditions.

To allay the bias potentially associated with attrition in the cohort, we applied inverse probability weighting (IPW), a recognized technique to compensate for selection bias [107]. IPW will be carried out by identifying early predictors of later loss to follow-up in a logistic regression model, which will provide a fitted probability of remaining in the cohort at the time of assessment for every cohort entrant. IPW will be applied to participants in all analyses, increasing the influence of individuals unlikely to self-select and correcting, to the extent possible from the attrition model, for selection bias.

### Population Attributable and Avoidable Risks

The population attribute and avoidable risks (PAR) associated with each risk and protective factor will be estimated using a Monte Carlo approach [108] that fully accounts for the risk/protective factor interaction, confounding and other adjustments, and mediation, if applicable, as is necessary to avoid bias and correctly estimate the standard error of the PAR estimates [109]. PAR will be computed for each respiratory condition observed during the clinical assessment and a dichotomized version of the FEV1 Z-score primary outcome, namely, the indicator that lies below the lower end of normal (ie, 5% centile at −1.64). Such an approach is made possible by the completeness of the data in the early cohort assessments and allows the indirect estimation of the prevalence of respiratory conditions in the full cohort under mild assumptions on the loss-to-follow-up mechanism (see below).

As missingness in early childhood data is minimal, we will use a singly imputed data set obtained from a full conditional specification of the covariate distribution using a discriminant function for categorical values, for our analyses. There will be no attempt to impute missing outcome values, if any, from the clinical assessments.

### Inferential Setting

All tests will be carried out at the 5% significance level, against two-sided hypotheses. Estimates will be reported as point estimates and 95% CIs. Standard descriptive quantities and simple regression results will be presented for all outcomes and main risk and protective factors. The main analysis will be handled through generalized additive models under a normal family with an identity link. Assumptions of residual normality will be checked visually and using standard distributional tests; departures from normality will be dealt preferentially with the selection of an alternative family and link, and, in case of variance behavior inconsistent with a known family, with a generalized additive model for location, scale, and shape [110]. The main analyses will be fully adjusted and attempt to present causal estimates, as indicated above. Analyses will be carried out using standard procedures from SAS version 9.4 and R version 3.x, and the study statistician will produce or supervise the production of custom code to obtain the causal parameter estimates and to carry out the Monte Carlo estimation procedure.

## Results

This study was funded in October 2017 and received ethical approval in May 2018. Data collection began in June 2018 and ended in November 2019, with a total of 466 participants recruited for submission of the paper. Collection and collation of chest x-ray data is still underway, and data analysis and expected results will be published by November 2020.

## Discussion

### Principal Findings

To the best of our knowledge, this is the first observational study to address the high burden of respiratory disease in Pacific youth aged between 18 and 19 years by estimating its causal relationship with factors in early life (risk and protective), which impose long-term detriments in lung function and are associated with the presence of respiratory illness as this population moves into young adulthood and nears the lifetime peak lung function. It is also at the time when the difference in hospitalization rates for any respiratory illnesses moves from being higher in males throughout childhood to being higher in females in adulthood.

Although utilizing the PIF cohort entails some loss in representativeness with regard to the Pacific population at large, it brings advantages by avoiding the costs and risks of establishing a new study de novo. Attrition must be acknowledged in utilizing this PIF cohort, largely because of (1) transient residential mobility by the age of 18 years and (2) in part because of logistical difficulties. However, as an analytical epidemiological study, the sample needs only be representative with regard to the effect of interest to be an internally valid study. Even if, in the proposed study, attrition interacts with the relationship between covariates and outcomes, selection bias will be allayed at the analysis stage by IPW and plausible causal relationships, revealed [111].

### Conclusions

This study will measure current lung function and assess the presence or absence of respiratory disease in the PIF study birth cohort, a group at increased risk of respiratory disease, which mainly resides in a region with a high prevalence of respiratory disease. Findings may be relevant for Māori, with approximately 8% of the original PIF cohort having Māori heritage, and Māori experiencing a similarly unacceptable high rate of respiratory illnesses in New Zealand. Moreover, this information can be directly used to formulate public health strategies to reduce future disease in this high-risk group for life, which will be relevant to the population as a whole.

## Abbreviations

COPD: chronic obstructive pulmonary disorder
ESS-CHAD: Epworth Sleepiness Scale for Children and Adolescents
FEV1: forced expiratory volume in 1 second
FVC: forced vital capacity
IPW: inverse probability weighting
LCQ: Leicester Cough Questionnaire
PAR: population attribute and avoidable risks
PIF: Pacific Islands Families
REDCap: research electronic data capture
SGRQ: St. George’s Respiratory Questionnaire
SIR: sliced inverse regression
